# Exploratory Hemodynamic and Hematologic Trajectories by Culture Status Among Neonates Evaluated for Infection †

**DOI:** 10.3390/children13091131

**Published:** 2026-08-24

**Authors:** Andra Rotea, Bogdan Rotea, Vlad Alexandru Meche, Mădălina Suba, Ovidiu Roșca, Bogdan Cerbu, Ileana Enătescu, Mirabela Adina Dima, Emil Radu Iacob, Adrian Gluhovschi, Daniela Iacob

**Affiliations:** 1 Doctoral School, Victor Babeș University of Medicine and Pharmacy, 300041 Timișoara, Romania; andra.alecu@umft.ro (A.R.); bogdan.rotea@umft.ro (B.R.); bogdan.cerbu@umft.ro (B.C.); 2 Methodological and Infectious Diseases Research Center, Department of Infectious Diseases, Victor Babeș University of Medicine and Pharmacy, 300041 Timișoara, Romania; madalina.suba@umft.ro (M.S.); ovidiu.rosca@umft.ro (O.R.); 3 Dr. Victor Babeș Infectious Diseases and Pneumononcology Hospital, 300310 Timișoara, Romania; 4 Department XIII, Discipline of Infectious Diseases, Victor Babeș University of Medicine and Pharmacy, 300041 Timișoara, Romania; 5 Discipline of Neonatology, Victor Babeș University of Medicine and Pharmacy, 300041 Timișoara, Romania; enatescu.ileana@umft.ro (I.E.); dima.mirabela@umft.ro (M.A.D.);; 6 Center for Multidisciplinary Research and Management in Pediatric Surgical Conditions, 300011 Timisoara, Romania; radueiacob@umft.ro; 7 Department of Pediatric Surgery and Orthopedics, Victor Babeș University of Medicine and Pharmacy, 300041 Timișoara, Romania; 8 Department XII, Discipline of Obstetrics and Gynecology, Victor Babeș University of Medicine and Pharmacy, 300041 Timișoara, Romania; adigluhovschi@yahoo.com

**Keywords:** neonates, suspected infection, culture status, blood pressure, platelet count, base excess, longitudinal cohort, exploratory analysis

## Abstract

**Highlights:**

**What are the main findings?**

- In this exploratory cohort, neonates with a positive database culture field had lower early blood-pressure values and more negative base excess than those with a negative field; the clearest longitudinal hemodynamic signal involved diastolic pressure, whereas the MAP interaction was not significant.
- Platelet counts were lower in the culture-positive group throughout the first three postnatal days, with no significant culture-by-time interaction, indicating persistent group separation rather than progressive deterioration.

**What are the implications of the main findings?**

- Repeated physiologic and laboratory measurements may help describe culture-status-associated trajectories, but these associations cannot be interpreted as sepsis-specific effects without clinical adjudication, standardized sampling, and treatment data.
- The findings are exploratory and require validation with organism-level culture adjudication, exact sampling times, standardized blood-pressure methods, inclusion of fatal cases, and adjustment for illness severity and treatment exposures.

**Abstract:**

Background: Neonates evaluated for infection undergo rapid physiologic transition, making isolated measurements difficult to interpret. Because a positive culture field does not by itself establish clinical sepsis, we explored whether physiologic and laboratory trajectories differed by recorded culture status during the first three postnatal days. Methods: This single-center prospective observational longitudinal study included 65 neonates with linked measurements on days 1, 2, and 3. The exposure was a binary culture field in the source database; specimen source, organism identity, and pathogen-versus-contaminant adjudication were unavailable. Group-specific medians were summarized, and gestational-age-adjusted linear models with patient-clustered robust standard errors tested culture status, time, and culture-status-by-time effects. Results: The cohort included 26 culture-positive and 39 culture-negative neonates. On day 1, the culture-positive group had lower median systolic blood pressure (48.5 vs. 57.0 mmHg), diastolic blood pressure (24.5 vs. 33.0 mmHg), and MAP (31.0 vs. 41.0 mmHg), more negative base excess (−7.65 vs. −3.60 mmol/L), and lower platelet counts (219 vs. 282 × 10^9^/L). Diastolic pressure rose in both groups and converged by day 3; the adjusted culture-status-by-time interaction was significant (*p* = 0.012). MAP increased over time, but its interaction was not significant (*p* = 0.244). Culture-positive status was associated with a lower day-1 platelet count (adjusted beta, −73.0 × 10^9^/L; 95% CI, −128.1 to −17.8; *p* = 0.010), without evidence of a differential platelet trajectory (interaction *p* = 0.623). CRP was retained as a descriptive outcome only because of lower-limit censoring. Conclusions: Recorded culture status was associated with selected early hemodynamic, metabolic, and hematologic differences in this small exploratory cohort. These associations cannot be considered sepsis-specific and require confirmation after clinical culture adjudication, standardized timestamping and blood-pressure measurement, inclusion of fatal cases, and adjustment for illness severity and treatment exposures.

## 1. Introduction

Sepsis is defined in adults as life-threatening organ dysfunction caused by a dysregulated host response to infection, an approach operationalized by the Sepsis-3 framework and its clinical criteria [1,2,3]. The same framework cannot simply be transferred to newborns. Contemporary pediatric criteria emphasize infection-associated organ dysfunction [4], yet neonatal sepsis still lacks a universally accepted, developmentally specific consensus definition [5]. Culture positivity supports microbiologic confirmation, but culture alone does not establish the clinical syndrome of sepsis without compatible illness, organ dysfunction, or adjudication of possible contaminants.

The contrast with adult sepsis is mechanistically important. Age-stratified transcriptomic studies demonstrate that neonatal and adult patients do not mount interchangeable host responses to sepsis [6], and experimental and clinical neonatal data show that postnatal age itself modifies the immune response [7]. In the first days after birth, developmental immunity, placental and maternal exposures, prematurity, and cardiovascular transition coexist with infection-related inflammation. Consequently, neonatal sepsis may present as an evolving organ-response phenotype whose timing and magnitude differ from adult disease.

Etiology also varies by timing and maturity. Group B Streptococcus and Escherichia coli remain major causes of early-onset disease, with a disproportionate burden of E. coli infection and mortality among preterm infants [8,9]. Late-onset sepsis in very-low-birth-weight infants has a different exposure context and microbial distribution [10]. Across these settings, neonatal sepsis spans subclinical infection through multisystem disease, and burden estimates depend strongly on case definition and care environment [11].

Early recognition is difficult because the clinical signs overlap with prematurity, respiratory disease, perinatal compromise, and normal adaptation. Complete blood count indices have limited sensitivity when interpreted in isolation [12,13]. Risk stratification models that integrate maternal factors and the newborn’s evolving clinical condition can reduce indiscriminate treatment, but they were developed principally for infants born at or beyond 34 weeks and do not replace bedside assessment [14,15].

The neonatal Sequential Organ Failure Assessment (nSOFA) was developed to quantify respiratory, cardiovascular, and hematologic dysfunction and predict mortality in preterm infants with late-onset infection [16]. Its prognostic performance has subsequently been examined in an independent late-onset cohort [17], in early-onset infection [18], within the first 72 h after birth [19], and across neonatal intensive care units [20]. These studies support the clinical relevance of dynamic organ dysfunction, but nSOFA is a prognostic score rather than a diagnostic test for sepsis, and its components are strongly influenced by neonatal maturity and treatment.

Inflammatory biomarkers require similar caution. Serial C-reactive protein (CRP) measurements are more informative than a single early result [21], and repeated low values have been used to support shorter antibiotic courses in selected neonates [22,23]. Nevertheless, CRP kinetics depend on sampling time, gestational age, perinatal inflammation, and noninfectious stress; an initially low value cannot reliably exclude early neonatal sepsis [24].

Platelet decline is a biologically plausible marker of infection-associated hematologic dysfunction. Culture-proven neonatal sepsis has been associated with lower platelet counts, and both severity and timing can vary with the organism and clinical context [25,26,27]. Blood pressure and acid–base status provide complementary views of circulatory adaptation, but neonatal hypotension has no single universally valid threshold. Maternal and intrapartum risk factors influence baseline infection probability [28], while extremely preterm infants with low blood pressure may also have impaired cerebral perfusion and receive highly variable antihypotensive therapy [29,30].

This exploratory study examined culture-status-associated changes in hemodynamic, metabolic, and hematologic variables during the first three days after birth. We evaluated whether recorded culture-positive and culture-negative groups differed at the initial assessment and whether their trajectories subsequently diverged or converged. We did not assume that culture positivity alone established clinical sepsis.

## 2. Methods

### 2.1. Study Design and Setting

We conducted a single-center prospective observational longitudinal study in the neonatal intensive care unit of the Dr. Victor Babeș Infectious Diseases and Pneumonology Hospital, Timișoara. Routinely recorded clinical and laboratory data from neonates evaluated for infection were assembled across three postnatal assessment windows. Reporting followed observational-study principles. Patients were prospectively enrolled under a predefined protocol.

### 2.2. Participants

Eligible participants were neonatal-unit admissions evaluated for suspected infection, of any gestational age, with documented gestational age and available clinical and laboratory data after birth. Exclusion criteria were major congenital anomalies, transfer before completion of follow-up, insufficient longitudinal data, or refusal of permission to use medical information. Death should not be treated as an automatic exclusion in an organ-dysfunction study. The source workbook contained 73 records at each of the three time points. None of the eligible neonates died during the study.

### 2.3. Timing and Measurements

Measurements were grouped into broad postnatal windows: 0–24 h (day 1), 24–48 h (day 2), and 48–72 h (day 3). Exact collection times within these windows were not available in the analytic database; therefore, measurements could not be confirmed to have occurred at comparable postnatal times across participants or culture-status groups. Variables included systolic blood pressure, diastolic blood pressure, MAP, peripheral perfusion index, arterial pH, base excess, lactate, blood glucose, CRP, white blood cell count, platelet count, and available respiratory-support indicators. Gestational age, birth weight, sex, Apgar scores, multiple pregnancy, and mode of delivery were recorded at baseline. The measurement of blood pressure was performed with a manual cuff, MAP was calculated, and no neonates received vasoactive drugs.

### 2.4. Culture Exposure

The exposure was the source-database field Culture, coded 1 for a recorded positive culture and 0 for a recorded negative culture. The analytic database did not contain information on specimen source, organism identity, number of positive bottles, time to positivity, sampling blood volume, repeat-culture results, or clinician adjudication of pathogens versus contaminants. Culture-positive records therefore could not be adjudicated as true infection, and the study does not equate culture positivity with clinical or culture-proven sepsis. Results are reported strictly as associations with recorded culture status.

### 2.5. Outcomes

All outcomes were analyzed exploratorily because no prospectively registered primary endpoint or analysis plan was available. Hemodynamic outcomes included systolic pressure, diastolic pressure, and MAP; secondary descriptive domains included platelet count, base excess, pH, lactate, CRP, white blood cell count, perfusion index, and glucose. The manuscript distinguishes the statistically supported diastolic-pressure interaction from the weaker MAP evidence.

### 2.6. Data Preparation

The baseline, day-2, and day-3 sheets were linked by exact patient identifier. Decimal-comma entries were normalized, nonnumeric markers were treated as missing, and clearly impossible single-variable values were set to missing without removing the participant. CRP values below the assay threshold were represented as one-half the threshold only for descriptive summaries. Because raw values below the threshold were unavailable, CRP was not used for inferential claims or treated as an exact continuous outcome in the revised interpretation.

### 2.7. Statistical Analysis

Continuous variables are reported as their median and interquartile range (IQR), and categorical variables as their count and percentage. Day-1 comparisons used the Mann–Whitney U test for continuous variables and the Fisher exact or χ^2^ test for categorical variables, as appropriate. Benjamini–Hochberg false-discovery-rate control was applied across the preliminary baseline screening set.

For repeated outcomes other than CRP, linear regression models included categorical time (day 1 as reference), culture-status group, culture-status-by-time terms, and gestational age. Patient-clustered sandwich standard errors accounted for repeated observations. The culture-status coefficient estimates the adjusted group difference on day 1; the joint interaction test evaluates whether subsequent changes differed by culture-status group. Estimates are presented with 95% confidence intervals. All tests were two-sided. Because of the small sample and incomplete information on treatment timing and illness severity, the models were intentionally parsimonious and are not interpreted as estimating a sepsis-specific effect. Analyses were performed in MedCalc 23.6.4 using auditable scripts; statistical significance was set at *p* < 0.05, while interpretation emphasized effect size, consistency, and multiplicity.

## 3. Results

### 3.1. Cohort Assembly

The workbook contained 73 records at each time point and 72 unique patient identifiers. After data cleaning, 71 unique baseline participants remained: 26 (36.6%) culture-positive and 45 (63.4%) culture-negative. Exact identifier linkage across all three days yielded 65 participants for the repeated-measures cohort, including 26 culture-positive and 39 culture-negative participants. Six culture-negative baseline participants were not included in the complete linked cohort.

### 3.2. Baseline Characteristics

In the linked cohort, median gestational age was 33 weeks (IQR, 29–34) among culture-positive neonates and 32 weeks (IQR, 29–36.5) among culture-negative neonates. Median birth weight was 1760 g (IQR, 1112.5–1985) and 1950 g (IQR, 1200–2755), respectively. The groups had similar sex distributions. The culture-positive group had lower day-1 systolic, diastolic, and mean arterial pressure, more negative base excess, and lower platelet counts (Table 1). CRP was numerically lower in the culture-positive group in the threshold-substituted day-1 summary; because of lower-limit censoring, this comparison is descriptive and is not interpreted as an exact concentration difference. None of the baseline screening comparisons remained significant after false-discovery-rate correction; the minimum adjusted q value was 0.072.

### 3.3. Hemodynamic Trajectories

Median systolic pressure increased from 48.5 mmHg on day 1 to 65.5 mmHg on day 3 in culture-positive neonates and from 57.0 to 68.0 mmHg in culture-negative neonates. Median diastolic pressure increased from 24.5 to 38.5 mmHg in the culture-positive group and from 33.0 to 39.0 mmHg in the culture-negative group. The gestational-age-adjusted day-1 difference in diastolic pressure was −7.12 mmHg (95% CI, −12.16 to −2.08; *p* = 0.006), and the joint culture-by-time interaction was significant (*p* = 0.012). The day-3 interaction coefficient was +8.84 mmHg (95% CI, 2.94–14.74; *p* = 0.003), consistent with the convergence of the groups by day 3.

MAP rose from 31.0 to 46.5 mmHg in culture-positive neonates and from 41.0 to 49.0 mmHg in culture-negative neonates. The adjusted day-1 culture coefficient was −5.13 mmHg (95% CI, −10.32 to 0.06; *p* = 0.053). MAP increased over time (joint time *p* = 0.003), but the culture-by-time interaction was not statistically significant (*p* = 0.244). These results indicate a general postnatal increase in MAP with an initially larger absolute deficit among culture-positive neonates, without strong evidence that the full MAP trajectory differed between groups (Table 2).

### 3.4. Hematological and Metabolic Trajectories

Platelet counts were lower in culture-positive neonates at each time point, with medians of 219, 186, and 204 × 10^9^/L on days 1, 2, and 3, compared with 282, 237, and 270 × 10^9^/L in culture-negative neonates. In the adjusted repeated-measures model, culture-positive status was associated with a 73.0 × 10^9^/L lower platelet count on day 1 (95% CI, −128.1 to −17.8; *p* = 0.010). There was no evidence of a differential platelet trajectory (interaction *p* = 0.623), supporting a persistent group separation rather than progressive divergence.

Base excess was more negative in culture-positive neonates throughout follow-up, with medians of −7.65, −4.70, and −4.90 mmol/L on days 1, 2, and 3, compared with −3.60, −3.40, and −3.40 mmol/L in culture-negative neonates. The adjusted day-1 group coefficient was −3.38 mmol/L (95% CI, −6.08 to −0.68; *p* = 0.014); the culture-by-time interaction was not significant (*p* = 0.290). Lactate did not show a consistent culture-group difference.

### 3.5. Inflammatory Markers and Exploratory Outcomes

The threshold-substituted CRP medians were 2.5, 3.75, and 2.5 mg/L in culture-positive neonates and 5.0 mg/L at each time point in culture-negative neonates. These values are presented descriptively only. Because many results were recorded below the assay threshold, the medians do not represent exact concentrations, and no diagnostic, protective, or sepsis-specific inference is made.

Glucose showed a statistically significant culture-by-time interaction (*p* = 0.005), driven by higher and more variable values in the culture-positive group on day 2. This pattern was unstable, plausibly treatment-sensitive, and not accompanied by a day-1 group difference; it should be considered hypothesis-generating only. White blood cell count, pH, and lactate did not show robust culture-specific trajectories in the preliminary models. Peripheral perfusion index was unavailable on days 2 and 3 and therefore could not be modeled longitudinally (Table 3).

## 4. Discussion

### 4.1. Principal Findings

In this exploratory longitudinal cohort, recorded culture-positive status was associated with lower arterial pressure and more negative base excess immediately after birth, together with lower platelet counts through day 3. Diastolic pressure showed the clearest dynamic signal: the groups differed at day 1 and approached one another by day 3. MAP increased in both groups, but its culture-status-by-time interaction was not statistically significant. Platelet counts remained separated without evidence of progressive culture-specific deterioration. These findings describe associations with recorded culture status, not the biology or causal effects of neonatal sepsis.

### 4.2. Relevance to Neonatal vs. Adult Sepsis

The findings are best interpreted as exploratory culture-status-associated trajectories rather than a validated organ-dysfunction phenotype. Adult Sepsis-3 defines sepsis through infection-associated organ dysfunction [1,2,3], whereas neonatal practice must also account for developmental immunity, prematurity, perinatal compromise, and cardiovascular transition [5,6,7]. The observed combination of lower pressure, more negative base excess, and lower platelet count is compatible with several clinical pathways. It cannot establish neonatal sepsis because clinical diagnostic criteria, culture source, organism identity, and contaminant adjudication were unavailable.

### 4.3. Interpretation in Relation to Organ Dysfunction

The nSOFA literature provides clinical context for dynamic respiratory, cardiovascular, and hematologic dysfunction in neonates [16,17,18,19,20]. However, nSOFA was neither reconstructed nor analyzed in this study, and the present results do not validate or test that score. Discussion of nSOFA is therefore limited to background context.

The blood-pressure findings may reflect a combination of infection-related vasodilation or myocardial impairment, prematurity, perinatal compromise, fluid management, vasoactive treatment, and normal cardiovascular transition. In extremely preterm infants, low pressure can accompany reduced cerebral blood flow, yet thresholds and treatment practices vary substantially [29,30]. Adjustment for gestational age reduced but did not eliminate the early group difference. Convergence by day 3 could represent clinical response to treatment, postnatal adaptation, regression to the mean, or selective missingness; without treatment timestamps and exact sampling times, a causal treatment effect cannot be inferred.

The persistent platelet separation is biologically and clinically plausible. Prior work has demonstrated frequent platelet decline during culture-proven neonatal sepsis and variation associated with organism and clinical context [25,26,27]. Platelet count is also the hematologic component of nSOFA, underscoring its relevance as a marker of critical organ dysfunction [16]. Nevertheless, the medians in our cohort generally remained above thresholds used for severe thrombocytopenia, and organism-level analysis was impossible. The result should therefore be interpreted as a relative hematologic difference rather than as evidence that all culture-positive neonates developed clinically important thrombocytopenia.

### 4.4. The CRP Paradox

The lower observed CRP values in culture-positive neonates should not be interpreted as biologically protective or diagnostically reassuring. Serial CRP studies show that an initial normal value has insufficient sensitivity to exclude neonatal infection, whereas repeated measurements over the next 24–48 h are more informative [21]. CRP-guided discontinuation strategies require selected populations and serial testing [22,23], while neonatal CRP kinetics remain sensitive to postnatal timing and noninfectious confounding [24]. In this database, many values were entered as below an assay limit, and the preliminary half-threshold substitution created an artificial cluster at 2.5 mg/L. Recovery of exact collection times and raw laboratory reports is essential before CRP is modeled as a diagnostic marker.

### 4.5. Strengths and Limitations

A principal strength of this study is the availability of repeated hemodynamic, acid–base, inflammatory, and hematologic measurements across the first three days after birth. Exact identifier linkage and patient-clustered modeling preserve the longitudinal structure, while effect estimates and CIs provide more information than isolated *p* values. The study also focuses on a clinically coherent question that can be answered without relying on the currently nonstandard composite scores in the workbook.

Several limitations directly constrain interpretation. First, the sample is small and single-center. Second, culture status was available only as a binary field; specimen source, organism, blood volume, time to positivity, antibiotic timing, and contaminant adjudication were unavailable. Third, broad 24 h windows and absent exact timestamps prevented confirmation that measurements occurred at comparable postnatal ages. Fourth, the blood-pressure technique, whether MAP was measured or calculated, and pre-measurement exposure to fluids, vasoactive drugs, respiratory support, and antibiotics must be verified from source records. Fifth, gestational-age adjustment does not eliminate confounding by birth weight, Apgar score, respiratory disease, illness severity, or clinical management. Sixth, exclusion of deaths may cause survivor bias and must be quantified by culture status. Seventh, six culture-negative infants were lost during exact longitudinal linkage, one duplicated identifier remains unresolved, and several variables had missing, censored, or implausible entries. Finally, multiple outcomes were explored without a prospective protocol. The associations cannot be interpreted as sepsis-specific or used for clinical decision-making.

### 4.6. Clinical and Research Implications

If confirmed in a clinically adjudicated cohort, repeated blood-pressure, acid–base, and platelet measurements may help characterize early trajectories among neonates evaluated for infection. The present results do not support diagnosis, risk prediction, or treatment decisions based on any single marker. A definitive study should prospectively specify sampling windows and blood-pressure methods, adjudicate pathogens and contaminants, include fatal cases, record treatment timing, reconstruct nSOFA only according to its published algorithm, and test whether trajectories add information beyond gestational age and baseline illness severity.

## 5. Conclusions

In this small exploratory cohort, recorded culture-positive status was associated with lower early blood-pressure values, more negative base excess, and persistently lower platelet counts. The clearest hemodynamic interaction involved diastolic pressure; MAP showed a general postnatal increase without a significant culture-status-by-time interaction. Because true infection was not adjudicated and key timing, treatment, blood-pressure-method, and fatal-case data were unavailable, these associations cannot be interpreted as sepsis-specific and require independent validation.

## Figures and Tables

**Table 1:** Baseline characteristics of the exact-linked longitudinal cohort.

Characteristic	Culture-Negative (*n* = 39)	Culture-Positive (*n* = 26)	Unadjusted *p*
Gestational age, weeks	32.0 (29.0–36.5); *n* = 39	33.0 (29.0–34.0); *n* = 25	0.376
Birth weight, g	1950 (1200–2755); *n* = 39	1760 (1112–1985); *n* = 26	0.203
Male sex, *n*/N (%)	20/39 (51.3)	14/26 (53.8)	1.000
Apgar score at 1 min	7.0 (6.0–8.0); *n* = 38	6.5 (4.2–7.0); *n* = 26	0.057
Systolic BP, mmHg	57.0 (50.0–65.0); *n* = 39	48.5 (43.5–57.0); *n* = 26	0.033
Diastolic BP, mmHg	33.0 (23.5–40.5); *n* = 39	24.5 (18.0–33.5); *n* = 26	0.012
Mean arterial pressure, mmHg	41.0 (31.0–47.5); *n* = 39	31.0 (27.2–41.8); *n* = 26	0.040
Lactate, mmol/L	3.17 (1.91–4.34); *n* = 36	2.48 (1.65–5.23); *n* = 26	0.716
Base excess, mmol/L	−3.60 (−5.38–−1.75); *n* = 38	−7.65 (−10.03–−2.82); *n* = 26	0.032
CRP, mg/L	5.00 (4.25–6.10); *n* = 38	2.50 (2.50–5.00); *n* = 23	0.007
WBC, ×10^9^/L	14.77 (9.57–19.80); *n* = 37	10.90 (9.43–13.67); *n* = 21	0.179
Platelets, ×10^9^/L	282.0 (202.0–320.0); *n* = 37	219.0 (154.7–274.8); *n* = 21	0.043

Data are shown as median (IQR); *n* unless otherwise indicated. Variable-specific denominators are shown because of missing data. CRP values below the assay threshold were represented as half the threshold for this preliminary table. *p* values are unadjusted; none remained significant after Benjamini–Hochberg correction across the baseline screening set (minimum q = 0.072). BP, blood pressure; CRP, C-reactive protein; WBC, white blood cell count.

**Table 2:** Culture-stratified trajectories during the first three days after birth.

Outcome	Time	Culture-Negative	Culture-Positive
Diastolic BP, mmHg	Day 1	33.0 (23.5–40.5); *n* = 39	24.5 (18.0–33.5); *n* = 26
	Day 2	35.0 (30.5–42.0); *n* = 39	33.5 (30.0–38.2); *n* = 26
	Day 3	39.0 (33.0–42.0); *n* = 37	38.5 (33.2–45.0); *n* = 26
Mean arterial pressure, mmHg	Day 1	41.0 (31.0–47.5); *n* = 39	31.0 (27.2–41.8); *n* = 26
	Day 2	42.0 (38.5–49.0); *n* = 39	41.0 (37.2–45.0); *n* = 26
	Day 3	49.0 (42.0–53.0); *n* = 37	46.5 (41.2–52.2); *n* = 26
Platelets, ×10^9^/L	Day 1	282.0 (202.0–320.0); *n* = 37	219.0 (154.7–274.8); *n* = 21
	Day 2	237.0 (196.6–316.7); *n* = 36	186.0 (134.3–238.0); *n* = 17
	Day 3	269.6 (188.2–340.8); *n* = 36	204.3 (144.6–271.3); *n* = 17
Base excess, mmol/L	Day 1	−3.60 (−5.38–−1.75); *n* = 38	−7.65 (−10.03–−2.82); *n* = 26
	Day 2	−3.40 (−6.15–−1.77); *n* = 36	−4.70 (−8.40–−3.30); *n* = 25
	Day 3	−3.40 (−5.30–−1.20); *n* = 37	−4.90 (−6.90–−2.10); *n* = 21
CRP, mg/L	Day 1	5.00 (4.25–6.10); *n* = 38	2.50 (2.50–5.00); *n* = 23
	Day 2	5.00 (5.00–11.50); *n* = 37	3.75 (2.50–8.68); *n* = 20
	Day 3	5.00 (4.00–5.00); *n* = 33	2.50 (2.50–5.58); *n* = 16

Data are shown as median (IQR); *n*. Exact patient identifiers were linked across days, but variable-specific missingness remained. CRP was subject to lower-limit censoring.

**Table 3:** Gestational-age-adjusted longitudinal models.

Outcome	Culture Coefficient at Day 1 β (95% CI)	P Group	P Time	P Interaction
Diastolic BP, mmHg	−7.12 (−12.16 to −2.08)	0.006	0.173	0.012
Mean arterial pressure, mmHg	−5.13 (−10.32 to 0.06)	0.053	0.003	0.244
Platelets, ×10^9^/L	−73.0 (−128.1 to −17.8)	0.010	0.263	0.623
Base excess, mmol/L	−3.38 (−6.08 to −0.68)	0.014	0.918	0.290
Glucose, mg/dL	−2.29 (−31.66 to 27.08)	0.878	0.820	0.005

Models included categorical time, culture-status group, culture-status-by-time terms, and gestational age, with patient-clustered robust standard errors. The culture-status coefficient estimates culture-positive minus culture-negative on day 1. P interaction is a joint test of day-2 and day-3 culture-status-by-time terms. Estimates are exploratory, not multiplicity-adjusted, and cannot be interpreted as sepsis-specific effects. CRP is excluded from inferential modeling because of lower-limit censoring.

## Data Availability

The data presented in this study are available on request from the corresponding author due to the fact that they are not publicly archived.
